# The congenital myasthenic syndromes: expanding genetic and phenotypic spectrums and refining treatment strategies

**DOI:** 10.1097/WCO.0000000000000736

**Published:** 2019-08-05

**Authors:** An E. Vanhaesebrouck, David Beeson

**Affiliations:** a Veterinary Department, University of Cambridge, Cambridge; b Nuffield Department fo Clinical Neuroscience, Neurosciences Group, Weatherall Institute of Molecular Medicine, John Radcliffe Hospital, University of Oxford, Oxford, UK

**Keywords:** congenital myasthenic syndromes, ephedrine, next-generation sequencing, postsynaptic, presynaptic, salbutamol, synaptic

## Abstract

Congenital myasthenic syndromes (CMS) are a group of heterogeneous inherited disorders caused by mutations in genes encoding proteins whose function is essential for the integrity of neuromuscular transmission. This review updates the reader on the expanding phenotypic spectrum and suggested improved treatment strategies.

**Recent findings:**

As next-generation sequencing is taken into the clinic, its use is both continuing to unearth new causative genes in which mutations underlie CMS and also broadening the phenotypic spectrum for known *CMS* genes. The number of genes in which mutations may cause neuromuscular transmission defects has now passed 30. The defective transmission may be part of an overall more complex phenotype in which there may be muscle, central nervous system or other involvement. Notably, mutations in series of genes encoding proteins located in the presynatic motor bouton have been identified. Rare cases of mutations in basal laminar proteins of the synaptic cleft are coming to light and additional mutations/phenotypic features have been located in some of the larger neuromuscular junction proteins such as AGRN and MUSK, where previously mutation screening by sanger sequencing was time consuming and costly. Finally, there are more reports of the beneficial effects of treatment with β2-adrenergic receptor agonists in patients, and the study of their action in disease models.

**Summary:**

Recent studies of the CMS illustrate the increasing complexity of the genetics and pathophysiological mechanisms involved. With therapy tailored for the underlying disease mechanism treatment, although incomplete, is usually life-transforming. However, treatment for newly identified conditions in which myasthenia is only one component within complex multisystem disorder will prove challenging.

## INTRODUCTION

Congenital myasthenic syndromes (CMS) are a group of rare inherited disorders of neuromuscular transmission [1,2]. The syndromes share the clinical feature of fatigable muscle weakness, but the age of onset, presenting symptoms, distribution of weakness and response to treatment vary according to the gene harbouring the mutation(s) and the underlying molecular mechanism that impairs signal transmission at the neuromuscular junction. Typical characteristics of many myasthenic disorders are ptosis, ophthalmoparesis, facial and bulbar weakness, and generalized muscle weakness presenting in the neonatal period or early childhood. However, symptoms can present in later childhood, adolescence or even adulthood and the pattern of muscle weakness vary. In a number of subtypes, there is marked limb-girdle weakness but the eyes and facial muscles are spared, and this can make differential diagnosis more challenging. Due to the more widespread adoption of next-generation sequencing the number of different genes implicated in CMS continues to grow and is now greater than 30 (Table 1). It is likely that mutations in some of these newly recognized genes will be exceptionally rare causes of CMS and will essentially remain isolated case studies, whereas others will warrant inclusion in standard genetic screening panels. Many of the more recently identified mutations causing CMS are in proteins whose expression is not restricted to the neuromuscular junction and, not surprisingly, mutations in these genes may often give rise to disorders with complex phenotypes with varying levels of involvement of impaired neuromuscular transmission.

In this review, we report on the expanding number of ‘causative-genes’ in which mutations that underlie impaired neuromuscular transmission have been identified, we update on the growing phenotypic spectrum for both new and better established CMS subtypes, and review recent thinking on treatment strategies. 

**Box 1:**
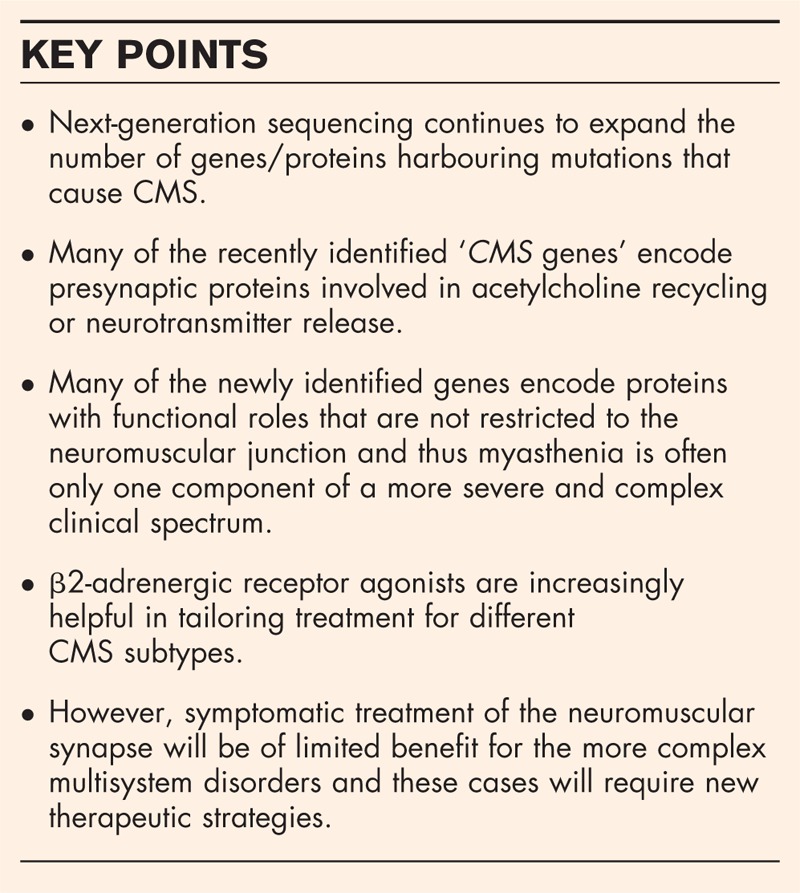
no caption available

## PRESYNAPTIC CONGENITAL MYASTHENIC SYNDROMES

It has become apparent that the presynaptic forms of CMS can be subdivided into two main categories; first, those involved in the synthesis and recycling of acetylcholine (ACh) and second, those involved in vesicle docking and transmitter release from the nerve terminal. Genes harbouring CMS-causing mutations in the ACh-recycling pathway include *SLC5A7*, *CHAT* and *SLC18A3*, which encode the high-affinity choline uptake transporter, cholineacetyltransferase and the vesicular ACh transporter, respectively [3–5]. Mutations in *CHAT* have long been established as a cause of a CMS characterized by episodic apneas and respiratory crises early in life that are often induced by infections or stress [4]. Between crises symptoms may be relatively mild and the number of life-threatening crises reduces with age so that by adolescence and adulthood occurrence is very rare. *SLC5A7* and *SLC18A3* CMS subtypes in general are more severe than choline acetyltransferase (CHAT)-CMS, and can lead to fatal foetal akinesia during pregnancy, and they share the same life-threatening apnoeic crises in early life. In addition, patients are reported to have marked ptosis, ophthalmoplegia, muscle fatigability, and in some cases [3,5] of both SLC5A7 and SLC18A3 CMS learning difficulties have been noted, which, in the absence of hypoxic damage, rarely if ever occurs in CHAT CMS. The reported phenotype for SLC5A7 CMS varies with many showing arthrogryposis/joint contractures at birth and limited survival, but other are relatively mild. Patients with mutations in the ACh recycling pathway respond to cholinesterase medication although in the severe cases the effect is minimal. It is of note that distal hereditary motor neuropathy type VII is caused by dominant mutations in *SLC5A7*[6,7], but surprisingly there appears to be little phenotypic overlap between the two disorders.

A second group of presynaptic syndromes features proteins involved in neurotransmitter release from the presynaptic nerve terminal. The soluble *N*-ethylmaleimide-sensitive factor attachment protein (SNARE) complex governs vesicle membrane docking, fusion and transmitter release from the presynaptic terminal. Synaptobrevin (*VAMP1*), syntaxin and synaptosomal-associated protein 25B (*SNAP25B*) form the core of the SNARE complex. Synaptotagmin 2 (*SYT2*) acts as a sensor detecting the influx of calcium following activation of voltage-gated calcium channels and interacts with the SNARE complex to trigger transmitter release. MUNC13 (*UNC13A*) is involved in assembly of the SNARE complex. Mutations in *VAMP1*[8,9], *SNAP25B*[10], *UNC13A*[11] and *SYT2*[12,13] have been found underlie syndromes in which synaptic transmission at the neuromuscular junction is impaired. However, SNARE-mediated membrane fusion is an essential feature of eukaryotic organisms and occurs at multiple sites and thus impaired neuromuscular transmission is often only likely to be one component within a wider multisystem disorder. For example, a de-novo SNAP25B mutation gave rise to a case of reduced quantal release from the motor nerve terminal, but also severe developmental delay, and ataxic gait and dysarthria. By contrast with the majority of CMS, SYT2 mutations give rise to a dominant disorder with features of a motor neuropathy and a neuromuscular transmission disorder resembling the autoimmune Lambert–Eaton myasthenic syndrome in which autoantibodies are directed against the presynaptic voltage-gated calcium channels. Like the Lambert–Eaton myasthenic syndrome, the syndromes arising from mutations in vesicle docking and neurotransmitter release mechanisms most commonly show increment of compound muscle action potential amplitude with high-frequency repetitive nerve stimulation. A more detailed review of the molecular mechanisms and electrophysiology of the presynaptic CMS is given in [14^▪▪^].

## BASAL LAMINA-ASSOCIATED SYNDROMES

Mutations in the collagen-like tail subunit (COLQ) that anchors the asymmetric form of acetylcholinesterase (AChE) to the basal lamina in the synaptic cleft of the neuromuscular junction are an established major cause of CMS. Some laminins subtypes are selectively expressed at the neuromuscular synapse and mouse models indicate a role in both synaptic structure and signalling but CMS resulting from mutations in laminin subunits are exceedingly rare. Single case reports have identified a homozygous mutation in laminin α5 (*LAMA5*) [15] and laminin β2 (*LAMB2*) [16]. In each case, the neuromuscular junction defect was reported in association with additional nonmyasthenic phenotypic features, for *LAMA5* it was myopia and facial tics, for *LAMB2* it is Pierson syndrome [17] characterized by chronic renal failure and neurodevelopmental problems. Collagen type XIII alpha 1 (COL13A1) is a single-pass type II transmembrane protein with a triple-helical collagenous ectodomain. It has main basic forms, one anchored in the plasma membrane via the transmembrane domain, and the second results from proteolytic cleavage of the ectodomain to produce a soluble product that interacts with components of the basal lamina. Mouse models have shown that loss of COL13A1 affects early maturation of neuromuscular junction structures on both pre and postsynaptic sides, and endplates remained small immature and fragmented [18]. In keeping with these studies of *COL13A1* function a report on 16 cases from 11 independent kinships found clinical presentation mostly at birth with hypotonia, and breathing and feeding difficulties [19^▪▪^]. Respiratory crises related to recurrent apnoea usually triggered by infections were common. Bilateral nonfatigable ptosis in adulthood and marked weakness of neck flexion were characteristic features. Patients respond to treatment with salbutamol and 3,4-diaminopyridine in their early years, but, whether treated or not, disease severity improves over time so that in some cases by adulthood muscle strength may be normal [19^▪▪^,^20^]. Studies of COL13A1 mutations emphasis the role of extracellular matrix proteins that are not part of the AGRN–LDL receptor-related protein 4 (LRP4)–MUSK–DOK7 signalling pathway in maturation of the mature synapse.

AGRN is a large extracellular matrix proteoglycan. It has several isoforms, and it is its neural isoform that plays a key role in maintaining synaptic structure through binding the LRP4 which in turn binds and activates MUSK [21]. Each of these three proteins is relatively large, with many exons, various splicing isoforms and multiple functional domains. The size and the number of exons within these genes made them challenging to screen using standard PCR and Sanger sequencing techniques. Next-generation sequencing has greatly facilitated mutation detection within these genes and led to the a broadening of the phenotypic spectrum for CMS due to either AGRN [22,23] or MUSK mutations including variants in MUSK giving rise to a late onset limb girdle CMS [24^▪^] isolated vocal cord paralysis [25] or pregnancy-associated respiratory failure [26]. It is likely that there will be increasing pick up of variants within these genes but defining pathogenicity will often require time-consuming and quite complex functional studies.

## GLYCOSYLATION PATHWAY CONGENITAL MYASTHENIC SYNDROMES

The asparagine-linked (N-linked) glycosylation pathway is a ubiquitous process present in all eukaryotic cells where there is sequential attachment of sugar moieties to a membrane lipid (dolichol) that are then transferred to a protein. The addition and processing of these glycans are crucial for the folding, assembly, stability and intracellular transport of proteins. Mutations in the genes which encode components of this pathway are often devastating causing severe multisystem disorders that may be fatal. However, a group of CMS, characterized by a limb girdle pattern on muscle weakness with little ocular or facial involvement, are found to have mutations in proteins that contribute to the early steps of this pathway [27–30]. Mutations within the same in genes, for example *DPAGT1*, can lead to either a severe multisystem disorder or a myasthenic disorder in which fatigable muscle weakness is the only presenting symptom. An article by Dong *et al.*[31^▪▪^] had shed light on what may underlie these very different manifestations. *DPAGT1* encodes dolichyl-phosphate (UDP-*N*-acetylglucosamine) *N*-acetylglucosaminephosphotransferase 1 and is the enzyme responsible for the first step in the assembly of the core glycan (Glc_3_Man_9_GlcNAc_2_) on the lipid dolichol. Crystallization of DPAGT1 in its apo state, when bound to UDP-GlcNac or tunicamycin and functional studies of DPAGT1 enzymatic activity showed that mutations the cause the multisystem disorder (CDG1j) are either null (frameshift or nonsense mutations) or affects catalytic activity, whereas those causing CMS tend to be located further from the catalytic site and may reduce protein expression/dimerization or modestly decrease catalytic activity. The neuromuscular junction is known to be very heavily glycosylated and it seems likely that it is sensitive to perturbations in function caused by modest impairment of the early steps of the N-linked glycosylation pathway. Uncovering the molecular structure of DPAGT1 should help predict the pathogenicity and severity of genetic variants detected in the *DPAGT1*[31^▪▪^].

## UPDATE ON TREATMENT STRATEGIES

**FIGURE 1:**
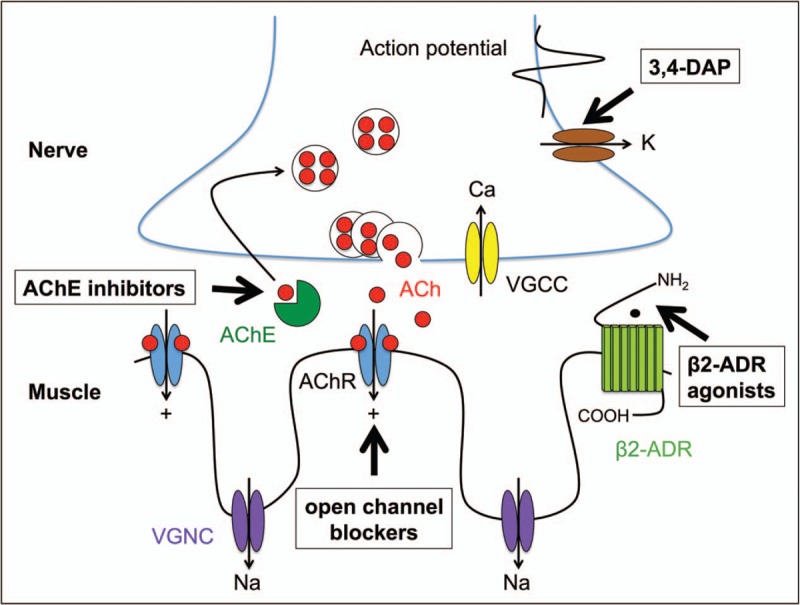
Diagrammatic representation for the sites of action for commonly used drugs for congenital myasthenic syndromes AChE, acetylcholinesterase inhibitors (neostigmine, pyridostigmine); 3,4-DAP, 3,4-diaminopyridine; open channel blockers (quinidine, fluoxetine); β2-ADR agonists, β2-adrenergic receptor agonists (salbutamol, ephedrine). VGCC, voltage-gated calcium channel; VGNC, voltage-gated sodium channel.

The current repertoire of drugs for use in CMS includes as follows:

Drugs that increase ACh release, such as potassium blockers (3,4-diaminopyridine); drugs that maintain high ACh concentrations within the synaptic cleft, such as AChE inhibitors (mainly pyridostigmine); β2-adrenergic receptor agonists (ephedrine, salbutamol); and ACh receptor (AChR) open-channel blockers (fluoxetine, quinidine) [1,32]. A diagrammatic representation of the neuromuscular junction and the site of action for these drugs are shown in Fig. 1. The response to treatment depends upon the subtype of CMS and the underlying pathogenic molecular mechanism. A number of recent reports have reviewed literature on treatment and provided potential algorithms for treatments strategies [32–34].

**FIGURE 2:**
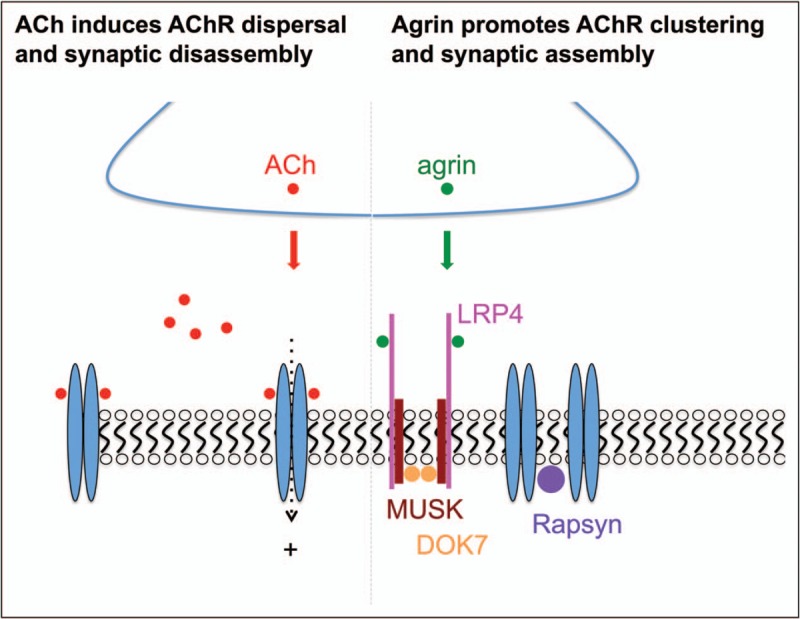
Pathways of synaptic disassembly and assembly at the neuromuscular junctions. Acetylcholine, released from the nerve terminal following a nerve stimulus, activates acetylcholine receptors. In addition to instructing muscle contraction, acetylcholine receptor activation is thought to disperse the tightly packed acetylcholine receptor away from the nerve terminal, which initiates synaptic disassembly. The agrin-induced acetylcholine receptor clustering pathway promotes synaptic assembly and thus counteracts the negative effect of acetylcholine on synaptic structure. Agrin is released from the nerve terminal, then binds LDL receptor-related protein 4, which activates MUSK and DOK7 and ultimately results in aggregation of postsynaptic acetylcholine receptors and maturation of the synaptic apparatus.

For many years reversible, competitive AChE inhibitors, such as pyridostigmine [35], were the mainstay of treatment for myasthenia. By blocking the action of AChE, the presence of ACh within the synapse is prolonged thus giving a greater probability of reaching the depolarization threshold for generation of the muscle action potential. Although effective for many CMS in others there was no clear response and in some it was positively harmful. Now, with greater understanding of the mutations and molecular mechanisms underlying CMS treatments can be tailored for the specific syndrome and depending on diseases severity and patient response this can include utilizing different combinations of the drugs. Pyridostigmine is quite clearly contraindicated for endplate AChE deficiency due to mutations in *COLQ*[36] as there is already a deficit of AChE function, and similarly in the dominant slow channel syndrome increasing the level and duration of ACh within the synaptic cleft is only likely to exacerbate the disorder. The use of AChR open channel blockers, fluoxetine or quinidine, can be remarkably effective for some slow channel mutations but the response is less marked for others. Adrenergic agonists, in particular ephedrine, have been used for the treatment of myasthenia, since the 1930s [37,38], but were largely replaced once AChE inhibitors were found to be effective in symptomatically treating myasthenia gravis [39]. Over the last 10–15 years, β2-agonists have re-entered as a mainstream option in treatment. Clearly an alternative to cholinesterase inhibitors was required for endplate AChE deficiencies, and in these patients a beneficial response to ephedrine was reported [40]. However, it was following the identification of DOK7 mutations as a major cause of CMS and their slow but remarkable improvement with β2-agonist medication that provided the impetus for its more widespread adoption. It is now apparent that not only DOK7-CMS but all the forms of CMS resulting from mutations in the AGRN–LRP4–MUSK–DOK7 signalling pathway for clustering AChR respond well to β2-agonists although the response for AGRN-CMS tends to be less pronounced. AChR deficiency patients on long-term anticholinesterase medication have also been found to benefit from β2-agonists. Long-term anticholinesterase has been shown to affect neuromuscular transmission and motor endplate fine structure [41], and so over time this treatment may become less effective. It appears that β2 agonists can alleviate these detrimental effects. Studies of ‘knock out’ mouse models of the neuromuscular junction identified that neurotransmission itself act to disperse AChR and disrupt synaptic structures and that this is countered by the input from the AGRN–LRP4–MUSK–DOK7 signalling pathway [42,43] (Fig. 2). A plausible way that β2 agonists have their beneficial effect is through enhancing the signal downstream that comes from the AGRN pathway and thus enhances and stabilize neuromuscular junction structure.

Little is known at the molecular level about how β2 agonists are exerting their effect at the neuromuscular junction, but a number of reports have begun to address this. Recently β2-adrenergic receptors were found to be present in higher densities at the postsynaptic membrane [44,45^▪^]. In addition, it is now thought that neuromuscular junctions might receive direct sympathetic innervation [44,46]. β2-adrenergic agonists do not have an immediate functional effects at the neuromuscular junction at doses clinically attainable [47] but fresh studies, using cell models, zebrafish or mouse models of myasthenia, all show that the β2-adrenergic receptor has a role in maintenance or enhancement of the postsynaptic structure of the neuromuscular junction [45^▪^,^48^,49^▪^].

## CONCLUSION

Many cases of CMS can be given effective symptomatic treatment with the drugs that are currently available once an understanding of the disease mechanism resulting from the mutation(s) is known. This often requires a balance between medication that directly enhances neuromuscular transmission and medication that helps maintain synaptic structure. However, clinical application of next-generation sequencing is revealing mutations in which myasthenia is only one component in a much wider disease phenotype. Symptomatic treatment of the neuromuscular junction is not always effective in severe cases of CMS and may not be appropriate for disorders in which myasthenia is the minor component in a multisystem disorder. We may now be reaching the time when it is apt to explore how novel gene therapies might be applied to these rare genetic disorders.

## Acknowledgements

We would like to acknowledge the support of the Wellcome Trust and the MRC for the funding that underlies this review. D.B. holds MRC programme grant MR/M006824. A.E.V. was a Wellcome Trust Clinical Training Fellow.

### Financial support and sponsorship

None.

### Conflicts of interest

There are no conflicts of interest.

## REFERENCES AND RECOMMENDED READING

Papers of particular interest, published within the annual period of review, have been highlighted as:

▪ of special interest
▪▪ of outstanding interest

## Figures and Tables

**Table 1:** Congenital myasthenic syndromes

Site of defect	Mechanism	Gene	Protein
Presynaptic	Defects in ACh recycling	*SLC5A7*	ChT
	Defects in ACh synthesis	*CHAT*	ChAT
	Defects in loading of ACh in synaptic vesicles	*SLC18A3*	VAChT
	Defects in synaptic vesicle docking, priming, fusing and exocytosis	*SNAP25B*	Soluble *N*-ethylmaleimide-sensitive factor attachment protein receptor 25
		*UNC13A*	Munc 13
		*SYB1/VAMP1*	Synaptobrevin-1/Vesicle associated membrane protein 1
		*SYT2*	Synaptotagmin-2
		*PREPL*	Propyl-endopeptidase-like
	Defects in axonal transport of proteins	*MYO9A*	Myosin IXA
Synaptic	Acetylcholinesterase deficiency	*COLQ*	Collagen-tail subunit of acetylcholinesterase
	Synaptic basement membrane defects	*COL13A1*	Collagen type 13 α1
		*LAMA5*	Laminin α5
		*LAMB2*	Laminin β2
	Defects in AChR clustering pathway	*AGRN*	Agrin
Postsynaptic	Reduced numbers of AChR (AChR deficiency)	*CHRNE(CHRNA1CHRNB1CHRND)*	AChR subunits
	Kinetic changes in AChR function (slow channel syndromes)	*CHRNA1CHRNB1CHRNDCHRNE*	AChR subunits
	Kinetic changes in AChR function (fast channel syndromes)	*CHRNA1CHRNDCHRNB1CHRNE*	AChR subunits
	Defects in AChR clustering pathway	*LRP4*	LDL-related protein 4
		*MUSK*	Muscle-specific tyrosine kinase
		*DOK7*	Downstream of kinase 7
		*RAPSN*	Rapsyn
	Defect in skeletal muscle voltage-gated sodium channel	*SCN4A*	Sodium voltage-gated channel α4
	Plectin deficiency	*PLEC*	Plectin
Pre + post	Defective glycosylation	*ALG2*	α-1,3-Mannosyltransferase
		*ALG14*	UDP-*N*-acetylglucosaminyltransferase
		*DPAGT1*	Dolichyl-phosphate *N*-acetyl-glucosaminephosphotransferase 1
		*GFPT1*	Glutamine-fructose-6-phosphate transaminase 1
		*GMPPB*	GDP-mannose pyrophosphorylase

ACh, acetylcholine; AChR, acetylcholine receptor; ChAT, choline acetyltransferase; ChT, choline transporter; COL13A1, collagen type XIII alpha 1; LRP4, LDL receptor-related protein 4; VAChT, vesicular acetylcholine transporter.
